# 急性T淋巴细胞白血病患者NOTCH1/FBXW7基因突变与CDKN2A/B基因缺失的特点及对预后的影响

**DOI:** 10.3760/cma.j.issn.0253-2727.2021.05.015

**Published:** 2021-05

**Authors:** 秋云 房, 其姗 郝, 晓媛 弓, 君 王, 庆华 李, 田 袁, 征 田, 凯奇 刘, 艳 李, 云涛 刘, 本法 宫, 迎 王, 辉 魏, 春林 周, 冬 林, 兵城 刘, 述宁 魏, 广吉 张, 建祥 王, 营昌 秘

**Affiliations:** 1 中国医学科学院血液病医院（中国医学科学院血液学研究所），实验血液学国家重点实验室，国家血液系统疾病临床医学研究中心，天津 300020 State Key Laboratory of Experimental Hematology, National Clinical Research Center for Blood Diseases, Institute of Hematology & Blood Diseases Hospital, Chinese Academy of Medical Sciences & Peking Union Medical College, Tianjin 300020, China; 2 天津医科大学肿瘤医院，国家肿瘤临床医学研究中心，天津市“肿瘤防治”重点实验室，天津市恶性肿瘤临床医学研究中心，天津市肿瘤免疫与生物治疗重点实验室 300060 National Clinical Research Center for Cancer, Key Laboratory of Cancer Prevention and Therapy, Tianjin's Clinical Research Center for Cancer, Key Laboratory of Cancer Immunology and Biotherapy, Tianjin Medical University Cancer Institute and Hospital, Tianjin 300060, China

急性T淋巴细胞白血病（T-ALL）是一类临床特征和预后异质性较大的疾病，遗传学异常是T-ALL的重要预后因素[1]。在T-ALL中，NOTCH1和FBXW7基因突变引起NOTCH1信号通路异常激活[2]，NOTCH1信号的组成性激活是T细胞转化中最重要的致癌途径[3]。文献报道T-ALL中NOTCH1突变的发生率约60％，FBXW7突变的发生率为10％～24％[4]。基因拷贝数变异（CNV）是ALL预后相关的另一重要遗传学异常，在T-ALL中发生率也很高，最常见的是染色体9p21中的CDKN2A/B基因（包含p16/INK4A和p14/ARF抑癌基因）的缺失，约70％的T-ALL患者发生该基因缺失[5]。Haydu和Ferrando等[6]报道NOTCH1突变和CDKN2A/B的CNV可以将T-ALL分为不同预后的亚组。我们应用二代测序及多重链接探针基因扩增（MLPA）方法对41例T-ALL患者分别进行了NOTCH1、FBXW7等基因突变以及CDKN2A/B、IKZF1、ETV6、RB1等13种基因缺失情况的检测，结合NOTCH1、FBXW7基因突变与CDKN2A基因缺失对T-ALL患者进行了预后分析。

## 病例与方法

1. 病例资料：2009年1月至2019年1月中国医学科学院血液病医院白血病诊疗中心收治的41例T-ALL患者纳入研究，初诊时均采用MICM模式（细胞形态学、免疫分型、细胞遗传学、分子生物学）确诊。其中37例患者在本中心接受统一方案的化疗（ChiCTR-TRC-00000397）[7]：诱导治疗采用VDC（L）P方案，在疾病达完全缓解（CR）后患者均接受早期强化治疗、再诱导强化治疗及维持治疗。37例患者中35例达CR，2例患者持续未缓解。37例患者的中位随访时间为16.4（5.4～135.1）个月。

2. 二代测序检测T-ALL患者NOTCH1及FBXW7基因的突变情况：留取初诊时的骨髓3～5 ml，应用QIAamp DNA试剂盒（德国Qiagen公司产品）提取骨髓单个核细胞基因组DNA，取1 µg DNA制备DNA全基因组文库。使用PCR引物扩增目的基因组（175个血液肿瘤相关基因），将目标区域DNA富集后，采用Ion Torrent测序平台进行测序。测序后原始数据利用人基因组数据库（hg19/GRCh37）、dbSNP、1000 genomes、HGMD、SIFT等数据库进行生物信息学分析，确定致病基因的突变位点。平均基因覆盖率99.0％，平均测序深度1200×。

3. MLPA：利用上述DNA标本，应用SALSA MLPA P335试剂盒（荷兰MRC-Holland公司产品）进行特定基因片段的扩增，所得产物置于ABI-3730基因分析仪（美国Applied Biosystems公司产品）进行毛细管电泳，最终结果应用Coffalyser^®^软件进行分析。SALSA MLPA P335试剂盒中包含与ALL发病相关的13种基因：CDKN2A/B、IKZF1、EBF1、PAX5、ETV6、BTG1、RB1、SHOX-AREA、CRLF2、CSF2RA、IL3RA、P2RY8。

4. 统计学处理：应用SPSS 17.0软件对数据进行统计分析。采用描述性分析及卡方检验对基因突变、基因缺失的临床特征进行分析及比较。采用Kaplan-Meier生存曲线及Log-rank检验进行生存分析。采用Cox回归分析法对预后的影响因素进行分析。*P*<0.05为差异有统计学意义。

## 结果

一、患者临床特征

41例T-ALL患者中男29例，女12例；中位年龄26（14～64）岁；中位WBC 41.61（1.18～613.31）×10^9^/L。在本中心接受治疗的37例患者中22例接受异基因造血干细胞移植（allo-HSCT）（移植组），未接受allo-HSCT（未移植组）患者15例。具体临床特征见表1。

**表1:** 41例急性T淋巴细胞白血病患者基因突变及缺失情况及与临床特征的关系

临床特征	总体（41例）	Notch1突变			FBXW7突变			CDKN2A/B缺失		
		是（17例）	否（24例）	*P*值	是（10例）	否（31例）	*P*值	是（20例）	否（21例）	*P*值
年龄［岁，*M*（范围］）	26（14～64）	26（14～52）	27（15～67）	0.97	27（20～64）	25（14～53）	0.49	26（14～50）	27（18～64）	0.06
性别（例，男/女）	29/12	11/6	18/6	0.53	9/1	20/11	0.11	18/2	11/10	0.01
WBC［×10^9^/L，*M*（范围）］	41.6（1.2～613.3）	44.6（1.2～222.8）	39.1（1.8～613.3）	0.57	41.6（4.3～145.4）	44.6（1.2～613.3）	0.51	53.5（4.3～613.3）	16.3（1.2～222.8）	0.14
移植（例，是/否）	22/15	11/6	11/6		6/4	16/11		12/7	10/8	
诊断分型（例）										
Pre-T	16	7	9		4	12		8	8	
Pro-T	11	3	8		2	9		3	8	
髓质T	9	3	6		2	7		5	4	
皮质T	5	4	1		2	3		4	1	

二、基因突变与基因缺失情况

41例T-ALL患者中24例（58.5％）具有NOTCH1和（或）FBXW7（N/F）基因突变。其中NOTCH1突变患者17例（41.5％），FBXW7突变患者10例（24.4％），同时合并两种突变者3例（7.3％）。

30例（73.2％）患者可检测到基因缺失，最常见的为CDKN2A/B基因缺失（48.7％），其中20例（48.7％）为CDKN2A基因缺失，14例（34.1％）为CDKN2B基因缺失。其他常见基因缺失（发生比例>5％）包括ETV6（22.0％）、EBF1（9.8％）、RB1（7.3％）、IKZF1（7.3％）；12例（29.3％）患者无任何基因缺失。

17例NOTCH1基因突变患者中有10例（58.8％）同时合并CDKN2A/B基因缺失，1例（5.9％）合并IKZF1基因缺失，4例（23.5％）合并ETV6基因缺失。10例FBXW7基因突变患者中有8例（80.0％）同时合并CDKN2A/B基因缺失，1例（10.0％）合并IKZF1基因缺失，3例（30.0％）合并ETV6基因缺失。3例同时具有NOTCH1及FBXW7基因突变者均合并CDKN2A/B基因缺失。7例（17.1％）患者不具有NOTCH1或FBXW7基因突变及任何基因缺失。

三、不同基因突变与基因缺失的预后分析

37例接受治疗的T-ALL患者3年总生存（OS）率为（46.7±12.9）％，3年无复发生存（RFS）率为（44.6±8.5）％。移植组患者生存明显好于未移植组：3年OS率分别为（68.2±9.9）％和（7.9±7.6）％（*P*<0.01），3年RFS率分别为（63.6±10.3）％和（9.2±8.7）％（*P*＝0.001）。

1. NOTCH1和FBXW7基因突变对T-ALL预后的影响：在患者总体和接受allo-HSCT患者中，NOTCH1基因突变对生存无明显影响（*P*>0.05），具有FBXW7基因突变者3年OS率和RFS率高于无此突变者，但差异无统计学意义（表2）。无论在具有NOTCH1基因突变患者中还是在FBXW7基因突变的患者中，接受allo-HSCT者生存明显好于未行移植者（表3）。

**表2:** NOTCH1、FBXW7基因突变及CDKN2A/B缺失对急性T淋巴细胞白血病预后的影响（％，*x*±*s*）

组别	患者总体			allo-HSCT患者		
	例数	3年OS率	3年RFS率	例数	3年OS率	3年RFS率
NOTCH1突变						
是	16	46.7±12.9	40.6±12.7	11	63.6±14.5	60.0±15.5
否	21	45.2±11.1	47.4±11.5	11	72.7±13.4	80.0±12.6
FBXW7突变						
是	9	71.4±17.1	64.8±16.5	6	83.3±15.2	83.3±15.2
否	28	40.6±12.7	38.5±9.5	15	66.7±12.2	64.3±12.8
N/F突变						
是	22	50.1±10.7	48.1±10.9	16	68.8±11.6	66.7±12.2
否	15	29.4±11.1	38.5±13.5	6	66.7±19.2	80.0±17.9
CDKN2A/B缺失						
是	20	33.4±11.1	35.2±10.8	12	54.5±15.0	54.5±15.0
否	17	58.8±11.9	60.0±12.6	10	90.0±9.5	88.9±10.5

注：allo-HSCT：异基因造血干细胞移植；N/F：NOTCH1和（或）FBXW7；OS：总生存；RFS：无复发生存。两组比较，*P*值均>0.05

**表3:** 异基因造血干细胞移植（allo-HSCT）对NOTCH1、FBXW7基因突变及CDKN2A/B缺失患者预后的影响（％，*x*±*s*）

组别	NOTCH1突变患者			FBXW7突变患者			N/F基因突变患者			CDKN2A/B缺失患者		
	例数	3年OS率	3年RFS率	例数	3年OS率	3年RFS率	例数	3年OS率	3年RFS率	例数	3年OS率	3年RFS率
allo-HSCT组	11	63.6±14.5	54.5±15.0	6	83.3±15.2	83.3±15.2	16	68.8±11.6	65.2±12.1	12	54.5±15.0	50.0±14.4
未移植组	5	0	0	3	0	33.3±27.2	6	0	0	8	0	0

*P*值		0.007	0.053		0.014	0.053		0.001	0.003		0.002	0.015

注：N/F：NOTCH1和（或）FBXW7；OS：总生存；RFS：无复发生存

具有N/F基因突变患者的生存略好于无此突变患者，差异无统计学意义（*P*>0.05）（表2）。具有N/F基因突变患者中，16例接受了allo-HSCT，移植患者的生存明显好于未行移植者（表3）。

2. CDKN2A/B基因缺失对预后的影响：具有CDKN2A/B基因缺失患者较无此缺失患者具有预后差的趋势，但差异无统计学意义（*P*>0.05）。尽管接受allo-HSCT患者的总体生存有改善，但CDKN2A/B基因缺失者较无此缺失者预后仍略差（表2）。allo-HSCT可以明显改善CDKN2A/B缺失的患者的生存（表3）。

3. NOTCH1基因突变与CDKN2A/B基因缺失的不同共存状态对T-ALL患者生存的影响：同时具有NOTCH1基因突变及CDKN2A/B基因缺失患者（10例）与单独具有NOTCH1基因突变患者（6例）3年OS率分别为（33.3±15.7）％和（66.7±19.2）％（*P*＝0.241），3年RFS率分别为（34.3±15.9）％和（50.0±20.4）％（*P*＝0.641），差异均无统计学意义。

20例CDKN2A/B缺失患者中，同时具有NOTCH1突变患者（10例）和单纯CDKN2A/B基因缺失患者（10例）3年OS率分别为（33.3±15.7）％和（33.3±15.7）％（*P*＝0.985），3年RFS率分别为（34.3±15.9）％和（30.0±14.5）％（*P*＝0.553），差异无统计学意义。

4. FBXW7基因突变与CDKN2A/B基因缺失不同存在状态对预后的影响：同时具有FBXW7基因突变和CDKN2A/B基因缺失患者（8例）与单独CDKN2A/B基因缺失患者（12例）相比，生存情况略好，3年OS率分别为（66.7±19.2）％和（16.7±10.8）％（*P*＝0.066），3年RFS率分别为（60.0±18.2）％和（16.7±10.8）％（*P*＝0.068）。

在FBXW7野生型患者中，CDKN2A/B缺失者（12例）与无此缺失者（16例）的3年OS率分别为（16.7±10.8）％和（58.8±11.9）％（*P*＝0.119），3年RFS率分别为（16.7±10.8）％和（52.9±12.1）％（*P*＝0.040）。接受allo-HSCT患者中，CDKN2A/B缺失者（7例）和无此缺失者（9例）的生存情况仍有显著差异，3年OS率分别为（28.6±17.9）％和（88.9±10.5）％（*P*＝0.024），3年RFS率分别为（28.6±17.1）％和（77.8±13.9）％（*P*＝0.067）。

## 讨论

T-ALL在成人及儿童急性白血病中发生率显著低于B-ALL，但有独特的临床及生物学特征[8]。随着化疗方案的改进及造血干细胞移植比例的升高，T-ALL的疗效得到了显著改善。尽管如此，T-ALL患者OS仍然明显差于B-ALL患者[9]。面对这些临床挑战，研究者们致力于探索T-ALL发病、耐药及复发等相关的分子学异常及其对T-ALL生存的影响，期待进一步优化T-ALL患者的预后分层系统，依据更精细的预后分层系统提供不同的治疗方案；发现更具体的治疗靶点，最终提高T-ALL患者的总体疗效。

T-ALL患者中NOTCH1及FBXW7基因突变发生率较高。NOTCH1和FBXW7基因突变可引起NOTCH1信号通路的异常激活，促进T-ALL的发生。另外，CDKN2A/B基因缺失的发生率很高，该基因缺失常与NOTCH1、FBXW7突变伴随发生，NOTCH1信号通路的组成性激活以及CDKN2A/B基因缺失的同时发生对T-ALL的发病起到了关键作用，两者对T-ALL患者的预后也具有一定的提示意义[5]。

Baldus等[10]报道126例T-ALL患者中NOTCH1和FBXW7基因突变的发生率分别为57％、12％，本组成人T-ALL患者NTOCH1基因突变发生率为41.5％（低于国外患者）。关于NOTCH1和FBXW7突变对T-ALL患者预后的影响，在国外的研究中结论并不统一[11]–[15]：Baldus等[14]发现，NOTCH1和FBXW7突变对成人T-ALL患者没有明显的预后意义，而在少数表达ERG/BAALC的低危组患者中，N/F突变型较野生型患者具有较好的生存趋势。在Asnafi等[15]关于成人T-ALL的研究中，N/F突变患者无事件生存及OS明显好于野生型患者，且发现FBXW7突变的存在明显提高了NOTCH1基因突变对T-ALL患者的预后意义。本组患者中，具有N/F突变的患者整体预后略好于无此类突变者；单独NOTCH1突变对患者的总体OS及RFS无明显影响，而单独FBXW7突变患者生存略好于无此突变者。虽然国内外关于FBXW7单独突变的研究较少，但综合现有结果可认为FBXW7突变对成人T-ALL患者的生存具有重要影响，可能提示预后良好。

本研究中成人T-ALL患者CDKN2A/B缺失的发生率明显低于国外研究中发生率（70％）[5]。但是生存分析中，T-ALL患者具有CDKN2A/B缺失提示具有预后较差的趋势，与Jang等[16]和Mirji等[17]的研究结果类似。尽管allo-HSCT可以改善伴CDKN2A/B缺失的T-ALL患者的OS，但在接受allo-HSCT的T-ALL患者中，伴CDKN2A/B缺失仍然是不良的预后因素。

研究显示75％的CDKN2A/B缺失患者合并NOTCH1或者FBXW7突变。我们结合NOTCH1、FBXW7突变及CDKN2A/B缺失情况进行了T-ALL患者的生存分析，结果发现NOTCH1基因突变的预后意义受到CDKN2A/B缺失的影响，两者同时存在OS也较差。FBXW7突变的存在可改善CDKN2A/B缺失患者的生存。

T-ALL的发生是一个复杂的过程，需要结合多种分子学异常对T-ALL患者的预后情况进行综合分析，基因突变检测和CNV分析十分重要。T-ALL患者应用单纯化疗的疗效并不理想，有条件者应首选allo-HSCT。本研究样本量较少，还需扩大样本量进一步验证研究结果。
